# P-978. Internal Medicine Resident Perceptions of the Barriers to and Facilitators of Optimal Inpatient Care of Persons who Inject Drugs: A Mixed Methods Study

**DOI:** 10.1093/ofid/ofae631.1168

**Published:** 2025-01-29

**Authors:** Rosemary C Bailey, Jessica S Tischendorf

**Affiliations:** UW Health - Madison, WI, Madison, Wisconsin; University of Wisconsin School of Medicine and Public Health, Madision, Wisconsin

## Abstract

**Background:**

Harm reduction counseling and HIV pre- and post-exposure prophylaxis (PrEP and PEP) can prevent infectious complications among persons who inject drugs (PWID). Hospitalizations are opportunities to provide these services, though these interventions occur infrequently for PWID admitted to our hospital. Little is known about internal medicine resident (IMR) perceptions of these interventions in the inpatient setting despite their frontline role in patient care in our institution.

**Figure:**
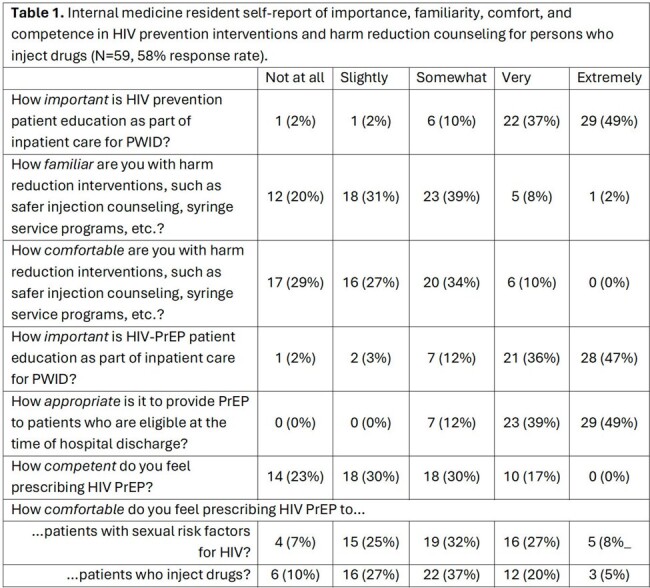

**Methods:**

In our mixed methods study we describe IMR perceptions of HIV prevention interventions and harm reduction counseling among PWID admitted to the University of Wisconsin Hospital. We conducted a survey examining current practices and perceptions. We explored the barriers and facilitators to optimizing these practices through semi-structured interviews. Transcripts were analyzed via consensus coding using the Systems Engineering Initiative for Patient Safety (SEIPS) model as a guiding conceptual framework. This framework describes the interactions of five components of complex work systems and their individual and overlapping influence on patient outcomes: 1) organization, 2) person, 3) physical environment, 4) task, and 5) technology and tools.

**Figure:**
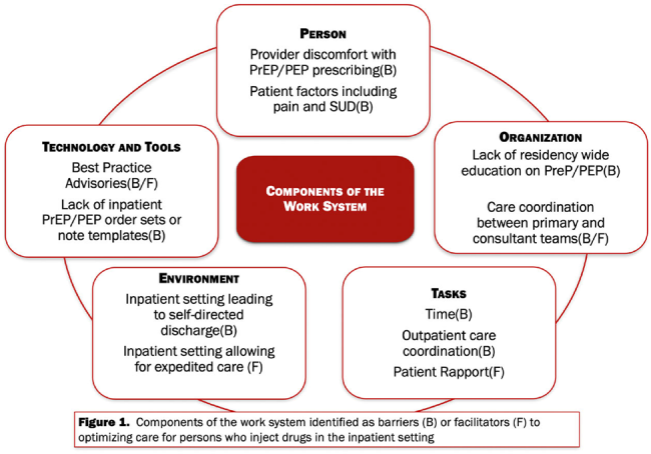

**Results:**

59 IMRs responded to the survey (58% response rate). Residents report offering HIV-PrEP or HIV-PEP to PWID at low frequency; 56% never offer PrEP and 64% never offer PEP. 20% or fewer IMRs report offering PrEP or PEP at least some of the time. IMRs report a high degree of importance and appropriateness of HIV prevention interventions; however, the majority lack comfort, familiarity, or competence in how to counsel patients or prescribe PrEP or PEP (Table 1). 15 residents participated in interviews, all of whom identified barriers and facilitators to these interventions relevant to each component of the work system (Figure 1).

**Conclusion:**

Despite recognizing the importance of HIV prevention services, including PrEP, PEP and harm reduction counseling, IMRs lack knowledge and comfort to do so. Barriers to optimal care span all components of the work system. Facilitators to optimal care and next steps include educational initiatives and EMR-based interventions with assessment of impact.

**Disclosures:**

**Jessica S. Tischendorf, MD, MS**, Merck: Grant/Research Support

